# Module Network Inference from a Cancer Gene Expression Data Set Identifies MicroRNA Regulated Modules

**DOI:** 10.1371/journal.pone.0010162

**Published:** 2010-04-14

**Authors:** Eric Bonnet, Marianthi Tatari, Anagha Joshi, Tom Michoel, Kathleen Marchal, Geert Berx, Yves Van de Peer

**Affiliations:** 1 Department of Plant Systems Biology, VIB, Gent, Belgium; 2 Department of Molecular Genetics, Ghent University, Gent, Belgium; 3 Unit of Molecular and Cellular Oncology, Department for Molecular Biomedical Research, VIB, Gent, Belgium; 4 Department of Biomedical Molecular Biology, Ghent University, Gent, Belgium; 5 CMPG, Department Microbial and Molecular Systems, KULeuven, Leuven, Belgium; University of Glasgow, United Kingdom

## Abstract

**Background:**

MicroRNAs (miRNAs) are small RNAs that recognize and regulate mRNA target genes. Multiple lines of evidence indicate that they are key regulators of numerous critical functions in development and disease, including cancer. However, defining the place and function of miRNAs in complex regulatory networks is not straightforward. Systems approaches, like the inference of a module network from expression data, can help to achieve this goal.

**Methodology/Principal Findings:**

During the last decade, much progress has been made in the development of robust and powerful module network inference algorithms. In this study, we analyze and assess experimentally a module network inferred from both miRNA and mRNA expression data, using our recently developed module network inference algorithm based on probabilistic optimization techniques. We show that several miRNAs are predicted as statistically significant regulators for various modules of tightly co-expressed genes. A detailed analysis of three of those modules demonstrates that the specific assignment of miRNAs is functionally coherent and supported by literature. We further designed a set of experiments to test the assignment of miR-200a as the top regulator of a small module of nine genes. The results strongly suggest that miR-200a is regulating the module genes via the transcription factor ZEB1. Interestingly, this module is most likely involved in epithelial homeostasis and its dysregulation might contribute to the malignant process in cancer cells.

**Conclusions/Significance:**

Our results show that a robust module network analysis of expression data can provide novel insights of miRNA function in important cellular processes. Such a computational approach, starting from expression data alone, can be helpful in the process of identifying the function of miRNAs by suggesting modules of co-expressed genes in which they play a regulatory role. As shown in this study, those modules can then be tested experimentally to further investigate and refine the function of the miRNA in the regulatory network.

## Introduction

MicroRNAs (miRNAs) are small endogenous regulatory RNAs, present in a wide variety of eukaryotic organisms. They are incorporated into an RNA induced silencing complex (RISC) that binds to sites of variable complementarity in target messenger RNAs, triggering their degradation and/or repressing their translation [1]. Evidence for the participation of miRNAs in cell growth, cell differentiation and cancer is currently piling up. Nearly half of the annotated human miRNAs map within fragile chromosomal regions, which are areas associated with various types of human cancers. Recent evidence indicates that miRNAs as well as the factors that participate in miRNA biogenesis may function as tumor suppressors and/or oncogenes [2]. According to the latest miRBase repository release [3], there are >700 human mature miRNA sequences identified with experimental support, while some computational studies expand this list to more than 1,000 [3], roughly equaling the number of transcription factors [4]. Computational and experimental studies have also predicted that between 30% and 100% of the human protein coding genes might be under the post-transcriptional regulation of miRNAs [5], [6]. It is not difficult to see that even by taking the most conservative values, the regulatory network induced by such a large number of regulators and targets is potentially extremely large. Furthermore, miRNAs do not act in isolation, but are part of a complex regulatory network, involving transcription factors, signal transducers and other types of regulatory molecules [7]. Reconstructing and analyzing such regulatory networks is thus a complex but crucial challenge to tackle.

Various algorithms exist to infer regulatory networks from expression data [8], [9], [10]. One of the most powerful methods, especially for eukaryotic organisms, assumes a modular structure of the underlying regulatory network, where a group of co-expressed genes is regulated by a common set of regulators (also known as the regulatory program) [10]. The regulatory program uses the expression levels of the set of regulators to predict the condition-dependent mean expression of the co-expressed genes. Thus, modules are composed of clusters of co-expressed genes together with their associated regulators. As a regulator can be associated with more than one module, the ensemble forms a module network. We have recently developed a novel algorithm which extends the original module network concept of Segal and co-workers [10] by using probabilistic optimization techniques which enable prioritization of the statistically most significant clusters of co-expressed genes and their candidate regulators [11], [12]. The main advantage of this algorithm is that it extracts more representative centroid-like solutions from an ensemble of possible statistical models, in order to avoid suboptimal solutions. By testing it on various biological datasets, we have shown that this approach generates more coherent modules, and that regulators consistently assigned to a module are more often supported by external sources of data [11], [12].

In this study, we have adapted our module network algorithm to take as input a heterogeneous dataset of both miRNA and messenger RNA (mRNA) expression data measured on the same samples. Multiple miRNAs are assigned as high-scoring candidate regulators for several modules, together with well-known transcription factors or signal transducers. A detailed analysis of three modules where miRNAs are selected as high-scoring regulators shows that this assignment is highly coherent with the module function and is also supported by various external sources of data. We have also validated one of those modules experimentally, showing that over-expression or inhibition of the miRNA assigned as a regulator changes significantly the expression of a selection of module genes, thereby confirming the inference of the algorithm. Those results corroborate module network inference as a robust and useful approach to gain more precise insights into miRNA function.

## Results

### Inference of a microRNA module network from expression data

**Figure 1:**
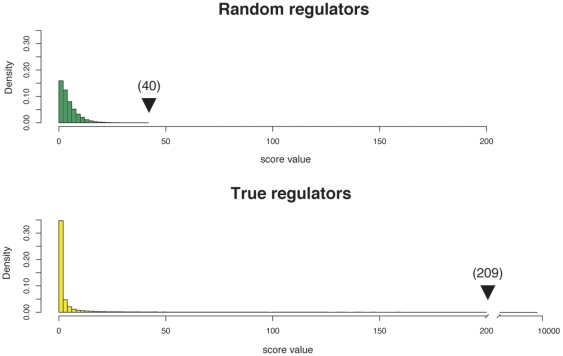
Random and real regulators score distributions. The histograms represent the distribution of randomly assigned (green) and true (yellow) regulators scores for the module network. The arrow for the random regulators represents the maximum score for randomly assigned regulators with the value indicated between brackets. The arrow for the true regulators represents the cutoff score value, with the raw value indicated between brackets.

The LeMoNe algorithm, starting from an expression data matrix and a list of candidate regulators, will produce a module network, composed of modules of co-expressed genes and their associated regulators. The algorithm is also clustering the conditions (columns) for each set of co-expressed genes, creating condition clusters. The list of regulators for a given module is ordered according to their individual score. This score only takes into account the differential expression of the regulator across the different different condition clusters, and not their absolute value. This way we can simultaneously evaluate and compare mRNA and miRNA candidate regulators, using the expression levels of each class of regulators. As input for our algorithm, we used a dataset composed of expression data measured on 89 tumor and normal tissue samples (representing 11 tumor classes) both for 11,833 messenger RNAs and 124 miRNAs [13]. Unlike previous attempts [14], [15], [16], our approach for the integration of miRNAs in the network is not based on miRNA target prediction, or a mixture between target prediction and expression data, but relies solely on expression data. The algorithm generated a set of 76 tightly co-expressed gene clusters, corresponding to a total of 2,987 genes. We calculated the GO enrichment for all the modules [17] and found a total of 44 clusters having at least one GO category enriched (p<0.05), for a total of 589 enriched categories (the complete list of modules and their GO categories is available as table S2). For the assignment of regulators, we compiled a list of 1,841 candidate regulators based on their GO annotation (either transcription factors or signal transducers), plus the list of 124 miRNAs. After the assignment of regulators we took a stringent cutoff corresponding to the top 2% most significant predicted regulatory interactions (Figure 1), obtaining a final set of 294 unique regulators (the complete list of module genes and regulators is available in table S1). Within this set, ten miRNAs were selected as regulators for a total of seven modules (Table 1). In order to assess the validity and the relevance of the inferred module network, we here present a detailed analysis of three modules, with an emphasis on the typical features of miRNA mediated regulatory modules. Those modules were selected based on their intrinsic interest, functional coherence and the high number of literature references discussing their putative function.

**Table 1:** List of modules where miRNAs have been selected as high-scoring regulators.

Module ID	Number of genes in the module	GO categories enriched for the module (p<0.05)	Regulators	Score
15	73	None	MAFB	3065
			NFIB	2774
			MSRB2	713
			IGFALS	464
			NR2C1	457
			CCL23	392
			MORF4L1	288
			FOS	249
			**let-7c**	231
			**miR-125b**	209
17	17	Digestion	NFIB	3785
		Proteolysis	HEYL	2361
		Lipid catabolic process	PLA2G1B	2164
			**miR-216**	1085
			CCRL2	780
			MC5R	327
			TRAF1	285
			CDX1	261
			DLX2	254
			E2F1	251
			IFNGR1	250
			CCR9	244
			TACR3	215
18	6	Immune response	HOXC5	1937
			HMGA1	1007
			**miR-142-5p**	863
			**miR-142-3p**	709
			HLA-DRB1	380
			CCL5	241
			AXL	215
			CXCL14	214
25	9	None	**miR-200a**	1786
			PPP1R1B	727
			GPR30	512
			PTGER3	456
			ZNF157	320
			GNB3	262
			GNG5	209
29	4	Actomyosin structure organization and biogenesis	PPP1R12B	7346
		Smooth muscle contraction	**miR-133a**	1216
		Smooth muscle fiber development	TSC22D1	1209
		Elastic fiber assembly	ANGPTL2	353
		Striated muscle development	**miR-145**	220
35	20	None	NFATC1	2827
			SH3BP1	2162
			FMNL2	699
			RASSF4	381
			GNAQ	290
			CTNNBIP1	288
			miR-181*	249
94	3	Folic acid metabolic process	**miR-10a**	253
		Regulation of Notch signaling pathway		
		Folic acid transport		

All regulators that are above cutoff are listed for each module, and ordered according to their decreasing score value. MicroRNA genes are highlighted in bold.

### MiR-133 and miR-145 are assigned as regulators of a smooth muscle actomyosin module

**Figure 2:**
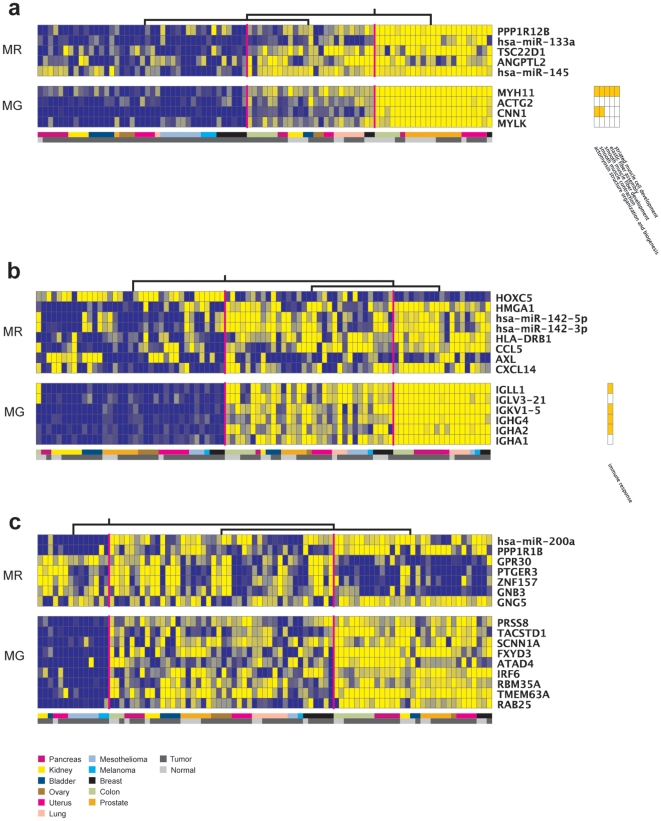
Modules 29 (a), 18 (b) and 25 (c) genes (MG) and assigned regulators (MR). Gene expression values are color coded, ranging from dark blue (low expression levels) to bright yellow (high expression levels). In each figure, columns represent a different sample. The color-coded bar at the bottom of the graph represents the tissue origin (see the legend), while the gray squares just below indicate whether the sample tissue was normal (light gray) or tumor (dark gray). The candidate regulators are ordered by decreasing score value (from top to bottom). The samples are grouped in leaves of homogeneous expression values, according to the hierarchical trees indicated on top of each figure. The orange boxes at the right of the figure indicate overrepresented GO categories (p≤0.05).

Module 29 is a small module composed of four genes and five assigned regulators (Figure 2a). The GO over-represented categories for this module are linked to smooth muscle development and actomyosin structure (Figure 2a and table S2). MYH11 encodes a smooth muscle myosin heavy chain family member. ACTG2 is a gamma 2 actin protein found in enteric tissues. The two other genes in the module (MYLK and CNN1) are well known regulators of the actin-myosin interactions. MYLK is the myosin light chain kinase, a dedicated calcium-dependent kinase that phosphorylates a specific site on the regulatory light chain of the myosin, enhancing its activity. MYLK is ubiquitous in all adult tissues with the highest amounts found in smooth muscle tissues [18]. CNN1 (calponin) is a calcium binding protein that inhibits the ATPase activity of the myosin in smooth muscle. The top regulator (PPP1R12B) selected for this module is a myosin phosphatase subunit. The myosin phosphatase is also a well known core regulator of the actomyosin pathway, inhibiting the myosin activity [18]. The second high scoring candidate regulator is a miRNA, miR-133, while the third regulator is the TGF-beta stimulated clone-22 member 1 (TSC22D1) gene, which encodes a leucine zipper domain protein, a member of the TGF-beta1 pathway which is involved in the regulation of transcription. The last regulators are ANGPTL2, a vascular endothelial growth factor, and another miRNA, miR-145.

The two miRNAs selected as regulators for this module clearly show a tightly positively correlated expression pattern with the module genes (Figure 2a). As most miRNAs have been characterized so far as negative regulators of gene expression, this suggest an indirect regulation between the miRNAs and module 29 genes. Recent studies reveal several likely candidate genes that could act as intermediate regulators between the miRNAs and module 29 genes. MiR-133, selected as the second best regulator for this module (Figure 2a), was recently shown to be a key regulator for skeletal muscle development and cardiac muscle hypertrophy [19], [20]. In those studies, miR-133 has been shown to directly regulate the SRF transcription factor. SRF is recognized as a vital factor for normal cytoskeletal and contractile cell activities and all the module 29 genes (*MYH11*, *CNN1*, *ACTG2*, *MYLK*) are known to be direct targets of SRF [21]. Those literature results support the hypothesis of an indirect regulatory link between miR-133a and module 29 genes via SRF. Most studies on SRF activity have so far characterized this factor as a transcriptional activator [21], but some results also suggest that SRF might act as a transcriptional repressor of its targets genes [22], [23], [24]. SRF mediated gene repression is not clear, but it might involve the recruitment by SRF of transcriptional silencers [23], [24]. If we hypothesize that SRF is repressing the transcription of module 29 genes, then the regulatory chain miR-133 – SRF – module 29 genes can explain the positive gene expression correlation that is observed in Figure 2a. The other miRNA selected as a regulator, miR-145 was recently shown to be an important regulator for smooth muscle cell fate [25]. This study [25] also shows that miR-145 is activating one of its direct targets, the myocardin (Myocd), which is a transcription factor well known to activate smooth muscle gene expression by interacting with SRF [26]. Thus we also have a regulatory chain miR-145 – Myocd – module 29 genes that can explain the pattern of expression observed in Figure 2a. Neither SRF nor Myocd are assigned as regulators or clustered together with the other module 29 genes. Unfortunately, the myocardin gene was not present in the microarrays used to produce the datasets [13]. The profile of the SRF transcription factor appears to correlate poorly with the expression of module 29 genes in our dataset (Data file S1), explaining why this gene could not be selected as a regulator. Several reasons could explain why the profile is divergent, like post-translational modifications or the fact that miRNAs act at the post-transcriptional level, possibly preventing the regulatory effect to be detected (by repressing the translation).

### MiR-142s are assigned as regulators of an immune response module

Module 18 is composed of six genes (Figure 2b), of which five encode immunoglobulins corresponding either to the heavy chain (IGHG4, IGHA2, IGHA1) or to the light chain (IGKV1-5, IGLV3-21), while IGLL1 is the surrogate light chain, a critical component of the pre-B cell receptor complex. Not surprisingly, we found the GO category immune response over-represented for this module (Figure 2b and table S2). All the module genes are known to be mostly expressed in developing and mature B-cells, revealing a coherent module [27]. Nine high scoring regulators were selected for this module. The top regulator is a homeobox gene, HOXC5. The HMGA1 gene is selected as the second best regulator for this module. High mobility group proteins (HMGA) regulate the activity of a wide variety of genes by changing the DNA conformation of their target genes. HMGA1 is known to co-activate transcription in B-cells and to be important for B-cells development [28]. The third and fourth candidate regulators are two miRNAs processed from the same precursor, miR-142-5p and miR-142-3p. The HLA-DRB1 gene belongs to the HLA class II beta chain paralogues. It is known to play a central role in the immune system by presenting peptides derived from extracellular proteins [29]. CCL5 is a chemotactic cytokine playing an active role in recruiting leukocytes to inflammatory sites [30]. AXL is a receptor tyrosine kinase that is transforming in fibroblast and hematopoietic cells, and is involved in mesenchymal development [31]. CXCL14 is a small cytokine belonging to the CXC chemokine family. This gene is chemotactic for monocytes and can activate these cells in the presence of an inflammatory mediator [32]. CXCL14 expression is reduced or absent from most cancer cells [33]. This module is probably linked to an immune response triggered by various tumor states. Such persistent pro-tumor immune responses are known to potentiate primary tumor development and malignant progression.

MiR-142s are preferentially expressed in hematopoietic tissues and their expression is regulated during hematopoiesis, suggesting a role in immune cells differentiation [34]. The transcription factor TCF12 is predicted as a target for miR-142-3p [35]. In a previous study, a combined dosage of the factors E2A, E2-2 and TCF12 was shown to be required for normal B-cell development. More precisely, TCF12 is important for the generation of normal numbers of pro-B-cells [36]. Because module 18 genes are expressed in developing B-cells, the regulation of TCF12 by miR-142-3p might be important for this process. Furthermore, we found conserved binding motifs for TCF12 for most module 18 genes (Data file S1), indicating that this transcription factor could be important for their regulation. Like for module 29 and SRF, the expression profile of TCF12 is highly divergent from the expression profiles of module 18 genes, explaining why this gene was not selected as module gene or regulator (data not shown).

### Mir-200a is a key regulator of a module involved in epithelial homeostasis

Module 25 is composed of nine genes (Figure 2c). SCNN1A (also known as ENaC) is the subunit alpha of the amiloride sensitive epithelial sodium channel, expressed in many epithelial tissues [37]. PRSS8 (prostasin) is a trypsinogen which regulates the activity of the epithelial sodium channel [38]. FDXY3 is a small membrane protein that is highly transcribed in tissues such as uterus, stomach and colon, and may function as a Na/K channel regulator [39]. TACSTD1, the tumor-associated calcium signal transducer 1, functions as a calcium-independent cell adhesion molecule [40]. Other genes like ATAD4 or TMEM63A are trans-membrane proteins of unknown function. RAB25 is a small GTP binding protein. RAB proteins have been involved in the regulation of vesicle trafficking [41]. The module top regulator is miR-200a. Regarding the other regulators, PPP1R1B is a phosphoprotein regulated by dopamine and cAMP, and is an inhibitor of the protein phosphatase 1. Besides its well-known role in the central nervous system, it is highly expressed in a variety of epithelial tissues where it might play a role in epithelial signaling and tumorigenesis [42]. GPR30 is a trans-membrane G protein coupled estrogen receptor [43], while PTGER3 is a G-protein coupled prostaglandin E2 receptor that is involved in various physiological processes and was shown to affect intracellular concentrations of Ca++ and cAMP [43]. ZNF157 is a zinc finger protein of unknown function while GNB3 and GNG5 are G proteins subunits involved in signal transduction. From the functions of these genes, we can conclude that most of the module 25 genes and regulators are likely involved in epithelial homeostasis, although we did not find any particular GO category enriched for this module. It is also worth noting that several of those genes are related to tumor progression [40], [41], [44].

**Figure 3:**
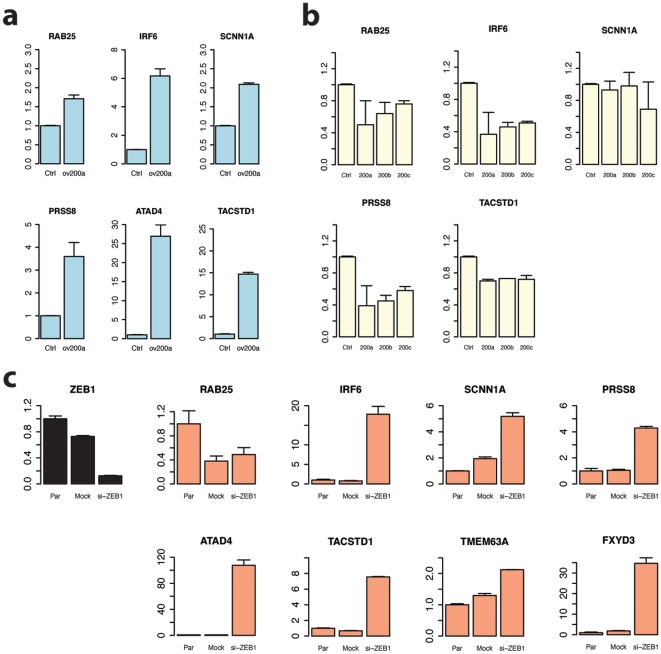
Validation of miR-200a as a regulator of module 25 genes expression. (a) Real time quantitative PCR (RT qPCR) analysis of the expression of module 25 genes *RAB25*, *IRF6, SCNN1A*, *PRSS8*, *ATAD4 and TACSTD1* and upon over-expression of miR-200a in MDA-MD-231 cells (mean ± standard deviation). The y-axis represents the relative mRNA expression value. miR-1 was used as the control (Ctrl), as it is not known to target any of the monitored genes (b) RT qPCR analysis of the relative expression for the genes *RAB25*, *IRF6*, *SCNN1A*, *PRSS8* and *TACSTD1* in MDA-MB-231 cells infiltrated with miR-200a,b,c antagomirs. The y-axis represents the relative mRNA expression value (mean ± standard deviation). miR-1 was used as the control (Ctrl) (c) RT qPCR analysis of the relative expression levels of module 25 genes *RAB25, IRF6, SCNN1A, PRSS8, ATAD4, TACSTD1, TMEM63A* and *FXYD3* in MDA-MB-231 cells where *ZEB1* is knocked down. The first barplot (black) shows effective repression of *ZEB1* levels upon transfection with the *ZEB1* specific siRNA. Par  =  parental cell culture, Mock  =  mock transfection, si-ZEB1  =  transfection with the ZEB1 specific siRNA.

MiR-200a, which was selected as the best candidate regulator for module 25, is a member of a miRNA family of five closely related miRNAs (miR-200a, miR-200b, miR-200c, miR-141 and miR-429). Recent publications show epithelial-specific expression of miR-200a and miR-200b [45], [46]. We designed a set of experiments to validate the role of miR-200a as a regulator of the expression of genes in module 25. MiR-200a was introduced in a human de-differentiated epithelial breast cancer cell line MDA-MB-231, known to express aberrantly low levels of miR-200a. The expression of six genes (*RAB25*, *IRF6, SCNN1A, PRSS8, ATAD4*, *TACSTD1*) out of nine belonging to module 25 was monitored using RT-qPCR (Figure 3). Without exception, the six monitored genes show a clear up-regulation upon exogenous expression of miR-200a (Figure 3a). The reverse experiment, inhibition of some members of the miR-200 family (miR-200a,b,c) in the MDA-MB-231 cells using antagomirs, resulted in the significant down-regulation of four out of five tested genes, (*SCNN1A* is not significantly down-regulated, *ATAD4* is not expressed in normal conditions in this cell line)(Figure 3b). Those results clearly show that miR-200a is a core regulator of module 25, most probably with other members of the miR-200 family.

**Figure 4:**
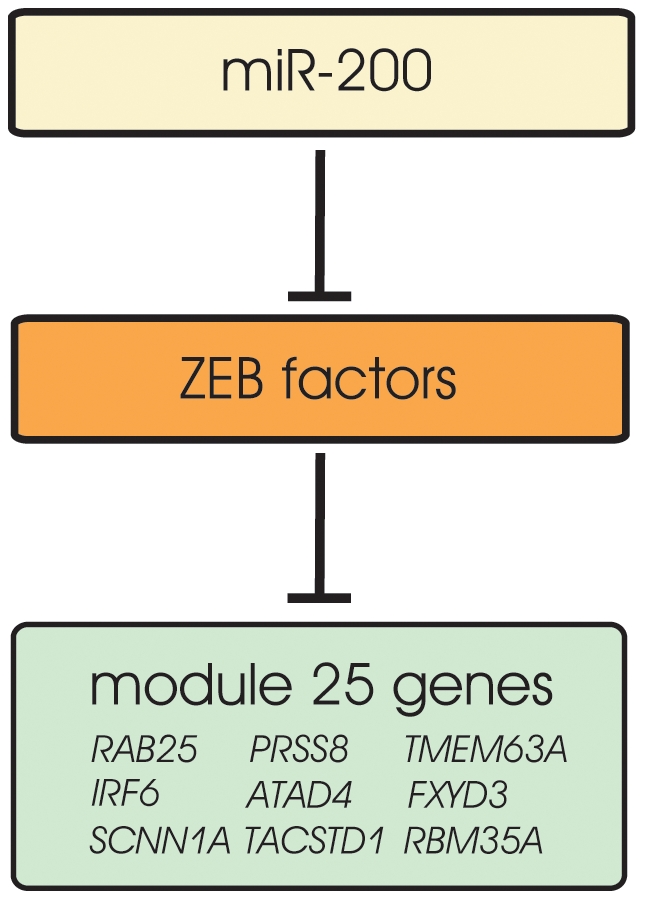
Module 25 hypothetical regulation model. MiR-200 genes repress ZEB factors, which in turn repress the expression of module 25 genes. The light yellow indicate genes assigned as regulators, the light green indicates module genes while the light orange indicates genes not assigned as regulators, but supported by literature (indirect regulation). This regulatory model support the positive correlation of the expression patterns between mir-200a and module 25 genes.

In this module, we observe again a clear positive correlation pattern between miR-200a and the module genes expression, suggesting an indirect regulatory circuit between miR-200a and module 25 genes (Figure 2c and Figure 3a and 3b). Recent experimental work showed that miR-200 family members directly target the transcription factors ZEB1 and ZEB2 [47], [48], [49]. These transcription factors are known as major transcriptional repressors of epithelial differentiation orchestrating epithelial mesenchymal transition (EMT) [50]. EMT is a process that drives epithelial cells from a polarized phenotype to a highly motile, non polarized mesenchymal phenotype and is known to occur in epithelial tumors giving rise to highly malignant cancer cells. The ZEB transcription factors have been functionally related to members of the miR-200 family via a double negative feedback loop, thus promoting EMT and cancer invasion [47], [51], [52]. We found conserved ZEB binding motifs for several module 25 genes (Data file S1), suggesting that ZEB factors could be the intermediate regulators between miR-200 and module 25 genes. To test this hypothesis, we down-regulated the ZEB1 transcription factor in MDA-MD-231 cells with a specific siRNA while monitoring the expression of eight module 25 genes (Figure 3c). All the genes show a strong up-regulation pattern, with the exception of the gene RAB25 (Figure 3c). Those results demonstrate that the ZEB1 factor is essential for the regulation of module 25 genes. Taken together, our experimental results (Figure 3) strongly suggest the existence of a regulatory chain between miR-200 and module 25 genes via the ZEB1 transcription factor (Figure 4). As both miR-200 and ZEB1 play important roles in EMT [47], [48], [49], [51], [52] we hypothesize that module 25 repression might contribute to the malignant EMT process in cancer cells.

## Discussion

MiRNAs have emerged quite recently as a new and important layer of regulation. Most of the studies so far have focused on their identification and on the detection of their targets. Several experimental studies have shown that at least some of them play key roles in various developmental and cellular pathways. Integrating miRNAs in regulatory networks is therefore of fundamental importance and should ideally be done taking into account the other types of regulatory molecules. So far, a few studies have proposed a computational strategy to infer miRNA mediated module networks [14], [15], [16]. These were mainly based on miRNA target prediction, or on a combination of target prediction and expression data. We have applied a robust and unbiased module network inference algorithm to a cancer-related expression data set of both mRNAs and miRNAs. In our approach, miRNAs were considered as candidate regulators, together with other types of regulators, like transcription factors and signal transducers. Even after applying a stringent cutoff, several miRNAs were retained as high scoring, statistically significant candidate regulators for various modules. Through an in-depth analysis of three of those modules, we showed that the assignment of specific miRNAs as regulators is supported by various external sources and is functionally coherent. Furthermore, we could show experimentally that a miRNA, assigned as the best regulator, is indeed a key regulator for the module genes expression. The number of miRNAs assigned in this study (10) might seem rather modest, but this number has to be evaluated with respect to the total number of miRNAs for which expression was measured in the samples (124). The ratio assigned per total number of miRNAs is equal to 8%, while the same ratio value is 15% for the ensemble transcription factors plus signal transducers (284/1841). The two ratio values are comparable and therefore we can reasonably expect a higher number of miRNAs assigned when an increased coverage of the miRNome expression landscape will be available.

Nevertheless, just as with other similar methods, care has to be taken for the interpretation of the inferred regulatory model. In particular, correlation of gene expression might not always indicate a direct interaction. Indeed, for the three modules we have investigated in detail, we have found an indirect regulation pathway between the regulator and the module genes. Furthermore, none of those indirect regulator genes were assigned by the algorithm in the regulation program or even clustered together with the module genes. As we could show for module 29 and the SRF transcription factor, the reason is because those indirect regulators expression profile differ significantly from those of the module genes. Various reasons can explain this divergence, for example the regulation might happen at the post-transcriptional level, or might be the result of post-translational modifications. Indirect regulators might of course complicate the interpretation of the results but they are to be expected, especially in higher eukaryotic organisms where regulatory networks are expected to be more complex [53].

Taken together, our results show that novel insights can be gained from a robust module network analysis of miRNA and mRNA expression data and support the view that at least some miRNAs have key regulatory roles in important cellular processes. Our approach has also the advantage of providing a direct view of post-transcriptional modifications through the integration of miRNAs, where mRNA expression alone might not be enough to reveal the existence of regulatory interactions. All three modules for which we did a detailed analysis in this study have each a coherent set of genes, involved in the same process and function. Furthermore, by connecting miRNAs to coherent modules, we believe that this approach can help to elucidate miRNA function and could efficiently drive experimental work towards the identification of key regulatory components in various processes. With the rapid proliferation of various techniques to measure with a high accuracy the levels of expression for hundreds of miRNAs, and the concomitant availability of mRNA expression data, it will be highly appealing to apply computational strategies like the one we describe here to expand our knowledge on global regulatory networks.

## Materials and Methods

### Expression data sets

We used a normalized cancer expression data set previously published [13]. We performed additional filtering steps to improve the quality of the input data set. Probesets with no known ensembl gene identifiers were discarded, as well as miRNA sequences that were not annotated as human miRNAs in the most recent miRBase release [3]. The final data matrix contained 11,833 genes and 124 miRNAs, for which expression was measured across 89 samples covering 11 different tumor classes.

### Module network inference

We used the LeMoNe algorithm to infer the module network [11], [12], [54]. In a first step, the algorithm is searching for a partition of genes into clusters of co-expressed genes. In a second step, the algorithm defines a regulatory program (a set of regulator genes) for each cluster. To avoid local optima traps in the first step, the algorithm uses a gibbs sampling approach for two-way clustering of both genes and conditions [54]. For a given input expression matrix, multiple clustering solutions are generated. For this study, we generated 30 different cluster solutions from the initial dataset. This ensemble of partially overlapping solutions is averaged to produce a set of tight clusters, representing subsets of genes which consistently cluster together in all solutions. The set of tight clusters is extracted using a graph spectral method [54]. For the second step, regulation programs are learned using a fuzzy decision tree model. The two-way clustering of the first step has also generated condition clusters (set of conditions having a similar mean and standard deviation) for each set of co-expressed genes. The condition clusters of a given module are first linked together in a hierarchical decision tree. Each node in the tree is defining a split between two sets of conditions (corresponding to low and high expression levels). Regulators are assigned to each node of the tree using a probabilistic score reflecting how well the expression levels of the regulator match the genes expression levels defined by the split value (for details about the mathematical model of the algorithm, see [11]). Just as for the gene clusters, multiple solutions are generated for the conditions clusters. Consequently, there are multiple decision trees and multiple regulators assigned for each node of each hierarchical tree. We adopt an ensemble approach again by summing the strength with which a regulator participates in each regulatory program for a given set of co-expressed genes. A global score is calculated, reflecting the statistical confidence of the regulator over all the nodes of all the hierarchical trees generated for the set of co-expressed genes [11]. For this study, we assigned up to 100 regulators for each node of each of the 100 hierarchical trees defined for each module. It is worth noting that by using a score that only takes into account the differential expression of a regulator across the different condition clusters, we can simultaneously evaluate and compare mRNA and miRNA candidate regulators. In the end, the set of regulators assigned to each cluster of co-expressed genes can be ranked according to their global probabilistic score and a cutoff level can be defined, keeping only very high-scoring regulators. In order to evaluate the statistical significance of the assigned regulators, a second set of randomly assigned regulators is generated along the set of “true” regulators (Figure 1). The complete list of modules together with their high-scoring regulators for this study is available in the table S1. The LeMoNe software package can be downloaded from our website, is open-source and free of charge for academics (http://bioinformatics.psb.ugent.be/software/details/lemone).

### Gene Ontology over-represented categories

For each module, we calculated GO enrichment using the BiNGO tool [17]. The complete list of GO categories enrichment for all the modules is available in the table S2.

### Transcription factor binding motifs

We used the ConTra [55] software tool to look for conserved TCF12 and ZEB motif binding sites in the promoter regions of module 18 and 25 genes. A multiple alignment of nine eutherian mammal species (*Bos taurus, Canis familiaris, Equus caballus, Pan troglodytes, Pongo pygmaeus, Macacca mulatta, Mus musculus and Rattus norvegicus*) and a specific position weight matrix were used to determine the conservation of the motif across all species.

### Cell culture

Human cancer cell lines were originally obtained from ATCC. MDA-MB-231 cells were maintained in Leibovitz's L-15 medium supplemented with 10% fetal calf serum (FCS), 100 µg/ml penicillin, 100 µg/ml streptomycin and 0.03% L-Glutamine. These cells were grown at 37°C without CO_2_ supply.

### miRNA repression and overexpression assays

MDA-MB-231 cells were seeded at 200.000 cells per well in 6-well plates in complete medium without antibiotics one day prior to transfection. The miRNA precursors and inhibitors as well as the positive and the negative control miRNAs were transfected at a final concentration of 25 nM using DharmaFECT 1 transfection reagent (ThermoSCIENTIFIC- Dharmacon) according to the manufacturer's instructions with the modification of using 8 µl of reagent instead of 6. The medium was refreshed after 18–24 hrs for the MDA-MB-231 cells and total RNA was collected 48 hrs post transfection. The negative control is Pre-miR™ miRNA Precursor–Negative Control #1, which does not target any known mRNA within the human or mouse transcriptome. The positive control is miR-1 Pre-miR miRNA precursor which has been shown to effectively downregulate the expression of twinfilin-1, also known as PTK9, at the mRNA level [56]. Validation of the downregulation of PTK9 was performed using a TaqMan® Gene Expression Assay (Assay ID: Hs00702289_s1). The control miRNAs and the qRT-PCR assay for human PTK9 were provided in the Pre-miR™ miRNA Starter Kit (Ambion Cat #AM1540).

### ZEB1 repression assay

MDA-MB-231 cells were seeded at 200.000 cells per well in 6-well plates in complete medium without antibiotics one day prior to transfection. ZEB1 siGENOME-SMARTpool was used for the downregulation of ZEB1 (ThermoSCIENTIFIC- Dharmacon, M-006564-02-0010), which consists of four SMART-selection designed siRNAs targeting one gene. The siZEB1 was dissolved in 1× siRNA buffer (ThermoSCIENTIFIC- Dharmacon, B-002000-UB) at a final concentration of 20 µM and was transfected at a final concentration of 25 nM using DharmaFECT 1 transfection reagent (ThermoSCIENTIFIC- Dharmacon) according to the manufacturer's instructions with the modification of using 8 µl of reagent instead of 6. As a negative control 1× siRNA buffer was used (MOCK transfection).

### Quantitative reverse transcription PCR (qRT-PCR)

Total RNA was extracted using Trizol (Invitrogen) according to the manufacturer's instructions with one modification; absolute ethanol was used in place of isopropanol. For the qPCR analysis cDNA synthesis was performed on 1 µg of total RNA using the iScript synthesis kit (BIO-RAD). The qRT-PCR for every gene was performed on 20 ng of cDNA in triplicate using the SYBRGreen I Master (Roche) or Probes Master (Roche) on a LightCycler®480 Real-time PCR System (Roche). The expression levels were determined using comparative quantification to the negative control and all quantification data were normalized against 2 reference genes, HMBS and TBP. The sequences of the RT-qPCR primers that were used are given in the data file S1.

## Supporting Information

Table S1 Complete list of module genes and regulators.
(0.28 MB XLS)

Table S2 Gene Ontology (GO) categories enrichment for each module.
(0.14 MB XLS)

Data file S1 Gene expression profile of MYH11, CNN1, ACTG2 and MYLK compared to SRF; TCF12 binding motifs for module 18 genes; ZEB binding motifs for module 25 genes; PCR primers.
(0.49 MB DOC)
