# Supplementary material for: Module Network Inference from a Cancer Gene Expression Data Set Identifies MicroRNA Regulated Modules
Source: PLoS One. 2010 Apr 14;5(4):e10162. doi: 10.1371/journal.pone.0010162 (PMC2854686; doi:10.1371/journal.pone.0010162)
Supplement: Data file S1 — Gene expression profile of MYH11, CNN1, ACTG2 and MYLK compared to SRF; TCF12 binding motifs for module 18 genes; ZEB binding motifs for module 25 genes; PCR primers. (0.49 MB DOC) [file pone.0010162.s003.doc]

**1. Gene expression profile for module 29 genes (MYH11, ACTG2, CNN1, MYLK) compared to the profile of the SRF transcription factor.**

**
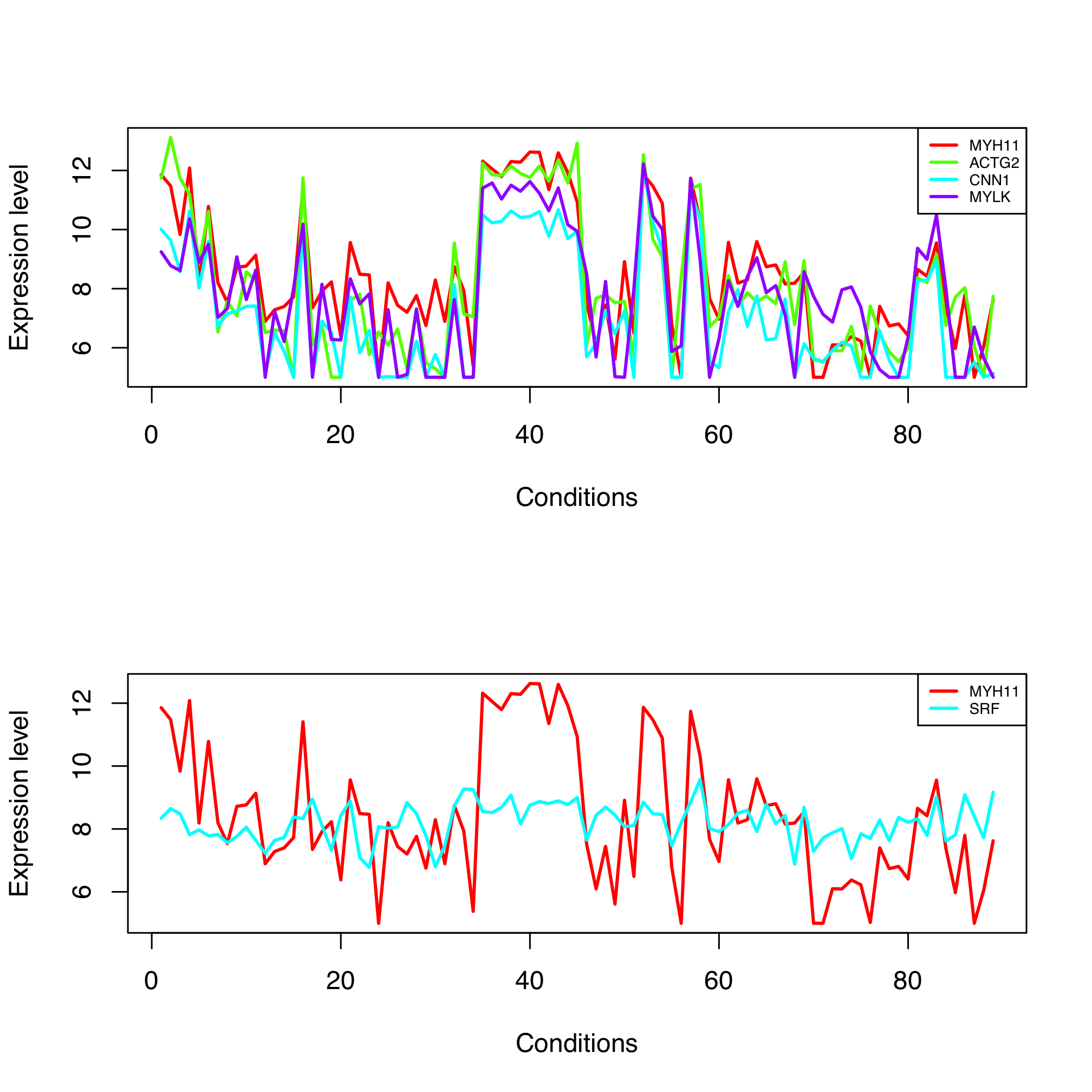
**

**2. TCF12 transcription factor binding motif for module 18 genes.**

**Human**: indicates if the binding motifs were found at least one in the human promoter.

**Conserved**: indicates if a conserved block was found in the 9 way mammalian alignments. 2sp(17) indicates that 17 aligned blocks for the motif were found in 2 species.

| **Gene name** | **Human** | **Conserved** |
| --- | --- | --- |
| IGLL1 | Yes | 2sp(17) |
| IGLV3-21 | Yes | 2sp(13) |
| IGKV1-5 | Yes | 0 |
| IGHG4 | Yes | 2sp(11) |
| IGHA2 | Yes | 3sp(4)  2sp(4) |
| IGHA1 | Yes | 2sp(2) |

**3. ZEB transcription factor binding motifs for module 25 genes.**

| **Gene name** | **Human** | **Conserved** |
| --- | --- | --- |
| TACSTD1 | Yes | 0 |
| PRSS8 | Yes | 4sp(8)  7sp(1) |
| SCNN1A | Yes | 4sp(6)  6sp(1)  8sp(2)  9sp(2) |
| FXYD3 | Yes | 4sp(1) |
| IRF6 | Yes | 4sp(3)  5sp(1)  6sp(1) |
| RBM35A | Yes | 4sp(3)  5sp(3)  6sp(6) |
| RAB25 | Yes | 4sp(3)  9sp(1) |
| ATAD4 | No | 0 |
| TMEM63A | No | 0 |

**4. Primer sequences used for the RT-qPCR experiments (5’ -> 3’).**

| **Gene** | **Forward** | **Reverse** |
| --- | --- | --- |
| Rab25 | TCTCCACCCGCACTGTGATG | GCCAGCTGTGTCCCAGATCTG |
| TACSTD1 | ATAACCTGCTCTGAGCGAGTG | TGAAGTGCAGTCCGCAAACT |
| IRF6 | CTGCGCTGTGCTCTCAATAAG | TCCTGGGTTAATGATCGAGCC |
| ATAD4 | TGGAGCAGTCGGGATTACA | TTGGGAGTGGATTTCTTTTTC |
| PRSS8 | CCAGCGAGCACCACAAGGAAG | GTAGCTGGGGTGGGGGATGAT |
| SCNN1A | ACGCTGGGCAACTTCATCTTC | AGGTTGGAGTTGTTCTTGTCA |
| FXYD3 | CGCCAATGACCTAGAAGATAA | CCCATGGCGCACAGAACC |
